# Evaluation of orthostatic dizziness and lightheadedness in older adults: symptoms not to be taken lightly

**DOI:** 10.3389/fneur.2026.1803374

**Published:** 2026-07-15

**Authors:** Svetlana Blitshteyn

**Affiliations:** 1 Department of Neurology, Jacobs School of Medicine and Biomedical Sciences, University at Buffalo, Buffalo, NY, United States; 2 Dysautonomia Clinic, Williamsville, NY, United States

**Keywords:** autonomic dysfunction, autonomic testing, orthostatic dizziness, orthostatic hypotension, orthostatic intolerance, patent care

## Abstract

Orthostatic dizziness and lightheadedness are frequent complaints in patients age 60 and above, whereas various common and uncommon etiologies need to be considered, including medication side effects, cardiovascular and metabolic causes and neurologic disorders. Autonomic dysfunction is a common etiology that warrants comprehensive medical and neurologic evaluations for identification of neurogenic orthostatic hypotension, prodromal Parkinson's disease, Lew body dementia, pure autonomic failure and others. In this article, key historical details, physical exam findings and diagnostic investigations for orthostatic dizziness are discussed. Patients' report of chronic and persistent orthostatic intolerance, even in the absence of objective orthostatic hypotension, should serve as a reliable and sufficient key feature should prompt an evaluation for autonomic and neurodegenerative disorders.

## Introduction

Orthostatic dizziness is a frequent complaint in adults age 60 years and older and represents a symptom that may be caused by heterogeneous etiology, including metabolic, cardiovascular and neurologic disorders. Orthostatic symptoms can arise from the interaction of age-related changes in cardiovascular and autonomic regulation, multiple medical conditions, polypharmacy, reduced baroreflex sensitivity and impaired cerebral autoregulation (1, 2). Consequently, evaluation of orthostatic dizziness in older patients requires a comprehensive, multidisciplinary approach integrating clinical history, medical and cardiovascular assessments, neurologic evaluation and targeted diagnostic testing.

## History

The initial evaluation relies on detailed symptom characterization, with particular attention to the phenomenology of dizziness and how it is described: usually, words such as “lightheadedness”, “presyncope” and “feeling faint” are used by the patient in reference to orthostatic dizziness and intolerance. Its temporal relationship to postural change whereas symptoms improve or resolve with sitting or supine position and are exacerbated by standing up, standing still or walking, needs to be established. Symptoms of movement, spinning, rotating, imbalance, disequilibrium or unsteadiness may suggest vertigo rather than orthostatic dizziness and point toward vestibular etiology. If vertigo is suspected, this should prompt a different diagnostic pathway than for orthostatic dizziness. In older adults, orthostatic symptoms may be delayed, non-specific, or poorly reproducible, necessitating careful correlation with posture, exertion, meals, hydration status, introduction of new medications and environmental stressors, such as heat.

Comorbid conditions and pharmacotherapies, including polypharmacy with its multiple and diverse interaction, which often includes orthostasis, are highly prevalent in people age >60 years. Hypertension, diabetes mellitus type 2, peripheral neuropathy, musculoskeletal problems, degenerative disease of the spine and joints and cardiovascular disorders are common contributors to orthostatic intolerance in this age group. Additionally, Parkinson's disease (PD), Lewy body dementia, pure autonomic failure and multiple system atrophy should be considered. A structured medication review and their side effects and interactions is essential, since antihypertensives, particularly diuretics and α-adrenergic blockers, nitrates, tricyclic antidepressants, antipsychotics, and dopaminergic agents, are well-established causes of orthostatic hypotension and orthostatic intolerance. Polypharmacy and recent medication changes are independent risk factors for orthostatic hypotension and falls (3).

## Physical examination

The most important test for evaluation of orthostatic dizziness is the measurement of orthostatic vital signs, specifically a stand test. Blood pressure and heart rate should be assessed after at least 5 min of supine rest and again after 3–5 min of standing although a 10-min stand test may be needed to detect delayed orthostatic hypotension (OH) if 5-min stand test is inconclusive (4–6). A sustained reduction in systolic blood pressure of ≥20 mmHg or diastolic blood pressure of ≥10 mmHg within 3 min of standing or on a tilt table test meets the diagnostic consensus criteria for orthostatic hypotension (5). Variations of OH include smaller, but symptomatic, reduction in SBP when the supine SBP is low (90–100 mm Hg) but drops well below this (5). In patients with supine hypertension, higher diagnostic thresholds, that is, SBP/DBP decline ≥30/15 mm Hg may be more appropriate because the magnitude of the orthostatic BP fall depends upon baseline BP (1). Delayed orthostatic hypotension—defined as a pathological blood pressure decline occurring after more than 3 min of standing—is increasingly recognized and clinically relevant (6). Delayed orthostatic hypotension may be associated with orthostatic intolerance, dizziness, presyncope, syncope and unexplained loss of consciousness or falls in patients >60 years of age (6).

Besides orthostatic blood pressure changes, heart rate responses provide insight into underlying mechanisms of orthostatic dizziness. A blunted chronotropic response suggests neurogenic orthostatic hypotension, which may be due to autonomic failure, whereas preserved or exaggerated tachycardia is more consistent with OH with compensatory mechanisms and may be due to hypovolemia or medication effects though the distinction between compensated orthostatic hypotension and uncompensated neurogenic orthostatic hypotension is now being questioned as it may exist on a spectrum (7). For patients with an orthostatic drop in blood pressure accompanied by an exaggerated compensatory increase in heart rate, ΔHR/ΔSBP ratio of < 0.5 supports neurogenic orthostatic hypotension (8). Comprehensive neurologic examination, including assessment of gait, parkinsonian features, cerebellar signs, and peripheral neuropathy, is critical in patients with orthostatic dizziness, given a strong association between orthostatic symptoms, neurodegenerative disease, and the risk of fall in older adults (3).

### Medical testing

Laboratory studies should include complete blood count and comprehensive metabolic panel to identify reversible causes and contributors to orthostatic dizziness, such as anemia, electrolyte disturbances, renal dysfunction, and metabolic disease (4, 5). Hemoglobin A1C and a 3-h glucose tolerance test to detect prediabetes and glucose intolerance may be beneficial. Vitamin and nutrient panels should be obtained. Electrocardiography, echocardiography and a cardiac stress test are indicated to rule out cardiac disease (4). These investigations are particularly important in older adults, in whom cardiovascular disease frequently coexists with orthostatic and exercise intolerance (9).

### Autonomic function tests

Formal autonomic testing, including tilt-table testing and autonomic reflex screen, which include Valsalva maneuver, heart rate variability to deep breathing, and sudomotor quantitative axon reflex testing, is reserved for patients with unexplained and persistent orthostatic intolerance, suspected neurogenic orthostatic hypotension, recurrent syncope and presyncope, neurogenic bladder and gastrointestinal dysmotility, including gastroparesis (10). A tilt table test may identify orthostatic hypotension, neurogenic orthostatic hypotension, delayed orthostatic hypotension, orthostatic hypertension, postural tachycardia or reflex vasovagal syncope (11). Symptom reproduction is also important, but reported symptoms and hemodynamic abnormalities often show poor concordance, especially in older population (12). Importantly, an unremarkable tilt table test does not exclude possible autonomic disorders, given both false positive and false negative test results (11). Lastly, transcranial Doppler in supine and tilted position may be used to identify symptomatic patients with relatively unremarkable vital signs who may have significant cerebral hypoperfusion via reduced cerebral blood flow velocity as measured in the middle cerebral artery (13). This test, if available, may provide objective evidence to subjective reports of orthostatic dizziness in patients whose stand test or tilt table test do not demonstrate significant abnormalities.

## Clinical features

Interpretation of orthostatic symptoms in adults age 60 and older requires recognition of age-related physiological changes, including reduced baroreflex sensitivity, arterial stiffening, and β-adrenergic alterations (12). These factors impair compensatory cardiovascular responses to standing and contribute to cerebral hypoperfusion even in the absence of diagnostic blood pressure thresholds or confirmed orthostatic hypotension. Conversely, some individuals demonstrate marked orthostatic hypotension with minimal subjective symptoms, suggesting reduced symptom perception leading to inability to safeguard against syncope and fall. Thus, when reported, orthostatic dizziness should prompt an evaluation of common autonomic disorders, and conversely, if orthostatic dizziness is not reported, specific questions need to be asked by the neurologist to determine if orthostatic dizziness is present. Beyond orthostatic dizziness, patients may report fatigue upon standing, generalized weakness, weak, heavy or cement-like legs while standing or walking, imbalance, unsteadiness, feeling faint or lightheaded, feeling unwell or tired, as well as other orthostatic features that are elicited or exacerbated by upright position (Table 1).

**Table 1:** Common symptoms and signs of orthostatic intolerance.

Symptoms	Signs
Orthostatic dizziness	Orthostatic hypotension
Orthostatic lightheadedness	Orthostatic hypertension
Chronic dizziness or lightheadedness	Orthostatic tachycardia
Feeling faint	Orthostatic pallor
Feeling weak	Orthostatic flushing
Orthostatic leg weakness	Dry mouth, eyes or skin
Heavy or “cement-like” legs while standing	Orthostatic restlessness that improves supine
Imbalance	Blood pooling on standing
Disequilibrium	Decreased consciousness
Unsteadiness	Loss of consciousness and fall
Orthostatic headache	Difficulty speaking on standing
Orthostatic nausea	Abnormal or slow gait
Cognitive impairment (“brain fog”)	Give-way weakness
Orthostatic visual disturbance	Visible fatigue on exam
Orthostatic shaking, trembling or vibration	Patient needing to sit or lie down
Orthostatic numbness or tingling	Patient constantly drinking water
Exertional fatigue	Orthostatic hyperventilation
Exercise intolerance	Fidgeting while sitting or standing

### Pathophysiology of orthostatic hypotension

Pathophysiology of orthostatic hypotension is diverse and multifactorial. In the elderly, aging coupled with diseases, such as DM and PD, results in a prevalence of 10%−30% (2). These conditions may cause baroreflex dysfunction with resulting combination of OH, supine hypertension, and loss of diurnal variation of BP (2, 12). Specific mechanisms for orthostatic hypotension may include peripheral noradrenergic sympathetic denervation, baroreflex dysfunction, chronotropic incompetence, decreased sympathetic response, impaired parasympathetic tone, vascular tone dysregulation, and altered inotropic efficiency (1, 2). Additionally, vascular stiffness from arteriosclerosis, cardiac changes with impaired diastolic filling and reduced preload as well as dehydration and reduced abilities of the kidney to conserve sodium and fluids, may be pathophysiologic factors in OH (1, 2, 12). Conversely, supine hypertension may lead to pressure diuresis and natriuresis as a mechanism contributing to orthostatic hypotension in autonomic dysfunction (2).

### Orthostatic hypotension and its risks

In patients aged 60 years and older, evaluation of orthostatic dizziness prioritizes careful clinical history with questions aimed at eliciting a history of orthostatic intolerance; repeated and extended orthostatic blood pressure measurements; rigorous medication and comorbidities assessment, and selective use of a tilt table test and detailed autonomic function testing. A comprehensive approach is essential, as orthostatic dizziness in this population may be multifactorial and associated with substantial morbidity, including falls, syncope, and cardiovascular events (1, 2). It may also signify neurodegenerative disorders, such as pure autonomic failure, PD-associated neurogenic OH, multiple system atrophy and Lewy body disease (2, 11).

OH is a risk factor for stroke (hazard ratio 2.0; 95% CI, 1.2–3.2) and why identifying OH as part of the physical exam, is necessary (14). Additionally, OH is a risk factor for falls in older adults (odds ratio 1.73, 95% CI 1.50–1.99), highlighting the clinical relevance of testing and treating OH to potentially reduce falls (15). OH is also a risk factor for cardiovascular disease, myocardial infarction, congestive heart failure and all-cause mortality (16). OH has been found to have a 40% risk of cognitive decline and 54% risk of progression from cognitive impairment to dementia over a 12-year follow-up period in dementia-free adults age 60 and older (17). These risks remained whether OH was symptomatic or asymptomatic. Additionally, in a study by Martinenz-Nunez et al. monitoring patients with hyposmia or REM-behavior sleep disorder for progression to Parkinson's disease, self-reported orthostatic dizziness and presyncope were identified in 118 patients age 60 and older as a risk factor (HR = 5.21, *P* = 0.01) for future cognitive decline in prodromal Parkinson's disease (16). The same study found patient-reported fatigue (HR = 9.32, *P* = 0.02) and constipation (HR = 7.81, *P* = 0.01) as predictors of cognitive decline (18). Importantly, these subjective predictors occurred without the objective nOH, which did not predict early cognitive decline in that study. However, this study had several limitations, including the use of specific subdomains of the SCOPA-AUT score that are not validated and no unified method for orthostatic testing. These and other limitations could have led to missed cases of delayed or initial orthostatic hypotension.

Accounting for all risks, patient reports of orthostatic dizziness and lightheadedness should be taken seriously, regardless of the presence of OH, and autonomic dysfunction needs to be considered in patients age 60 and older. These complaints, in conjunction with unexplained fatigue and constipation, may point to prodromal Parkinson's disease, especially if accompanied by decreased sense of smell or rapid eye movement behavior sleep disorder. Importantly, patient's reports of orthostatic dizziness, fatigue and constipation—i.e., subjective symptoms—should be taken as reliable and sufficient to activate evaluation for possible prodromal PD (18). Thus, neurologists should specifically ask patients whether they experience orthostatic dizziness, fatigue and constipation as part of the comprehensive neurologic evaluation.

### Subjective-objective mismatch

**Figure 1:**
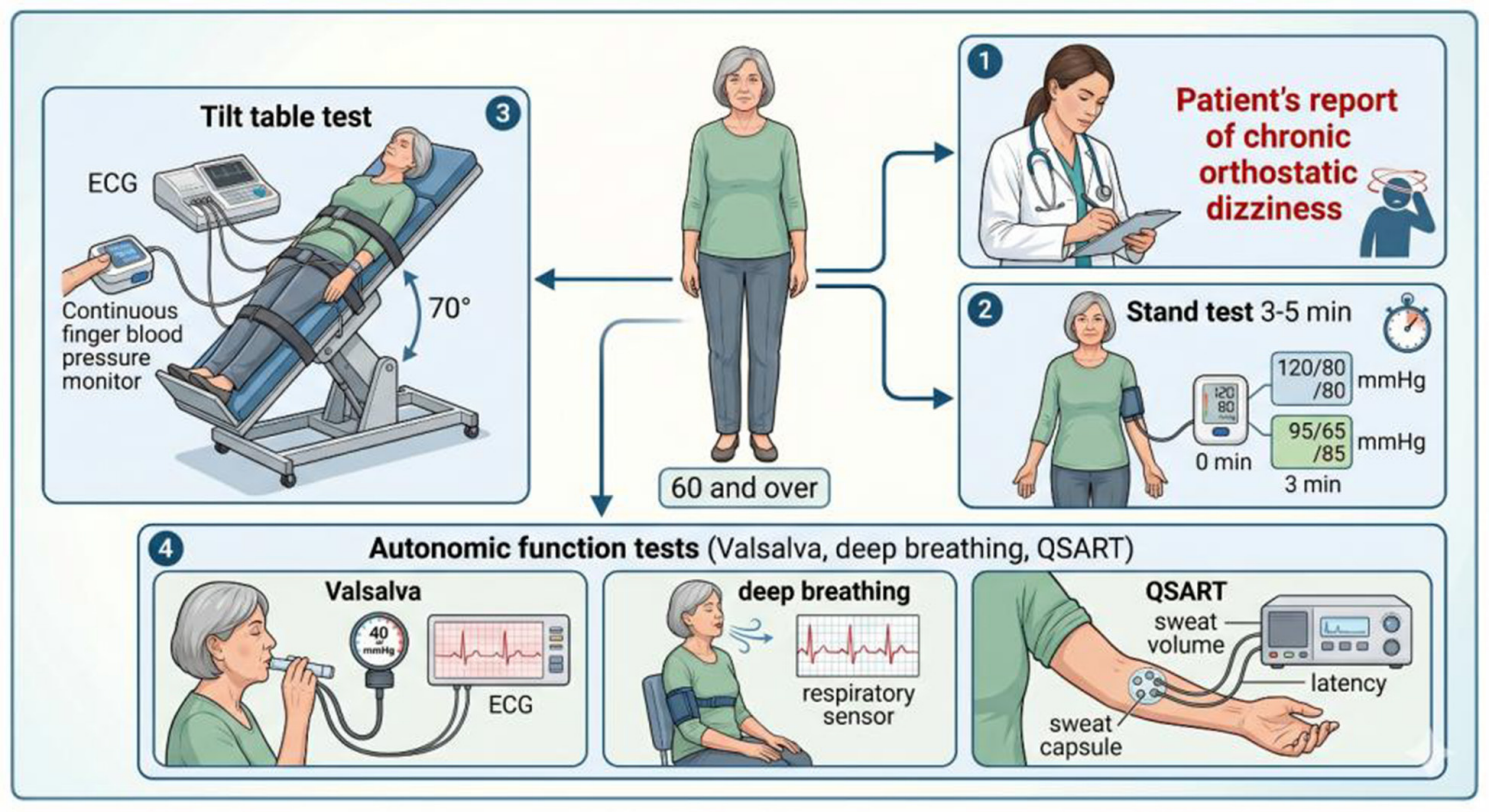
Autonomic assessment of chronic orthostatic dizziness.

Autonomic dysfunction and self-reported autonomic symptoms as part of dysautonomia are common among patients with neurologic and non-neurologic disorders (19). In the absence of objective vital signs abnormalities on a stand test or abnormal tilt table test, patients complaining of orthostatic dizziness and fatigue face significant diagnostic challenges because autonomic dysfunction is often not diagnosed as such (20, 21). Other non-neurologic causes, such as medication side effects, anxiety or deconditioning, are assumed, often erroneously, as an explanation for the patient-reported symptoms if autonomic testing fails to confirm significant abnormalities. Neurologic education consistently highlights the fundamentals of clinical medicine, which underscores detailed history and patient's reported symptoms as the most important and diagnostically valuable features, often overriding the unremarkable or minimally abnormal diagnostic testing (22). Importantly, patients' report of chronic orthostatic dizziness and lightheadedness should prompt an evaluation for possible autonomic disorder with a stand test, followed by a tilt table test and, if needed, complete autonomic function testing (Figure 1).

Many patients with common autonomic disorders, such as postural orthostatic tachycardia syndrome and post-COVID dysautonomia as part of Long COVID, are often not diagnosed with autonomic dysfunction or have subjective-objective mismatch when their autonomic function tests are deemed unremarkable or demonstrate minimal findings despite self-reported significant autonomic symptoms (21). Over-reliance on objective diagnostic tests, in conjunction with dismissal or false attribution of patient's complaints of orthostatic dizziness or lightheadedness in clinical practice, has led to undiagnosed dysautonomia or misdiagnosis with other, often psychiatric, disorders such as generalized anxiety, panic disorder, somatization or functional neurologic disorder (23). Similarly, over-reliance on objective diagnostic tests, especially when these tests lack complete autonomic function testing with transcranial Doppler, in clinical research presents a barrier to broadening the therapeutic landscape for dysautonomia. As the study by Martinez-Nunez et al. clearly illustrates, reports of orthostatic dizziness and fatigue in certain subsets of patients should not only be taken seriously as evidence of autonomic dysfunction, but may also indicate significant risk factors for disease course and progression (18). Developing validated scoring scales utilizing integrated approach that incorporates both subjective reports and objective diagnostic tests in patients with suspected autonomic dysfunction would be essential to improving both clinical care and research of autonomic dysfunction. Validated and easily accessible diagnostic modalities that assess cerebral perfusion during postural change are needed to provide objective measurement to subjective patient reports of chronic orthostatic dizziness and lightheadedness.

## Conclusion

Orthostatic dizziness and lightheadedness are common complaints in patients age 60 and older that require careful and detailed medical and neurologic evaluation to identify possible etiologies, rule out cardiovascular and metabolic disorders, and assess for possible neurodegenerative conditions that may present with orthostatic dizziness. Sometimes patients with orthostatic dizziness may face a diagnostic challenge when their complaints are not taken seriously, or when their objective autonomic tests return unremarkable or minimally abnormal. The subjective-objective mismatch and frequent misdiagnosis of autonomic dysfunction with other disorders, most commonly psychiatric, may result in diagnostic and therapeutic delay compromising favorable patient outcomes. Training neurologists how to recognize and manage autonomic dysfunction is imperative to improving medical care of patients presenting with orthostatic dizziness and lightheadedness.
